# NiftyPAD - Novel Python Package for Quantitative Analysis of Dynamic PET Data

**DOI:** 10.1007/s12021-022-09616-0

**Published:** 2023-01-09

**Authors:** Jieqing Jiao, Fiona Heeman, Rachael Dixon, Catriona Wimberley, Isadora Lopes Alves, Juan Domingo Gispert, Adriaan A. Lammertsma, Bart N. M. van Berckel, Casper da Costa-Luis, Pawel Markiewicz, David M. Cash, M Jorge Cardoso, Sebastién Ourselin, Maqsood Yaqub, Frederik Barkhof

**Affiliations:** 1 Centre for Medical Image Computing, Department of Medical Physics and Biomedical Engineering, University College London, London, UK; 2 School of Biomedical Engineering and Imaging Sciences, King’s College London, London, UK; 3 Radiology and Nuclear Medicine, Amsterdam Neuroscience, Amsterdam UMC, Vrije Universiteit Amsterdam, De Boelelaan 1117, Amsterdam, Netherlands; 4 Edinburgh Imaging, Queen’s Medical Research Institute, University of Edinburgh, Edinburgh, UK; 5 BarcelonaBeta Brain Research Centre, Pasqual Maragall Foundation, Barcelona, Spain; 6 Dementia Research Centre, Queen Square Institute of Neurology, University College London, London, UK

**Keywords:** NiftyPAD, PET, Pharmacokinetic analysis, Reference input-based modelling, Python package

## Abstract

Current PET datasets are becoming larger, thereby increasing the demand for fast and reproducible processing pipelines. This paper presents a freely available, open source, Python-based software package called NiftyPAD, for versatile analyses of static, full or dual-time window dynamic brain PET data. The key novelties of NiftyPAD are the analyses of dual-time window scans with reference input processing, pharmacokinetic modelling with shortened PET acquisitions through the incorporation of arterial spin labelling (ASL)-derived relative perfusion measures, as well as optional PET data-based motion correction. Results obtained with NiftyPAD were compared with the well-established software packages PPET and QModeling for a range of kinetic models. Clinical data from eight subjects scanned with four different amyloid tracers were used to validate the computational performance. NiftyPAD achieved $R^2>0.999$ correlation with PPET, with absolute difference $\sim 10^{-2}$ for linearised Logan and MRTM2 methods, and $R^2>0.999999$ correlation with QModeling, with absolute difference $\sim 10^{-4}$ for basis function based SRTM and SRTM2 models. For the recently published SRTM ASL method, which is unavailable in existing software packages, high correlations with negligible bias were observed with the full scan SRTM in terms of non-displaceable binding potential ($R^2=0.96$), indicating reliable model implementation in NiftyPAD. Together, these findings illustrate that NiftyPAD is versatile, flexible, and produces comparable results with established software packages for quantification of dynamic PET data. It is freely available (https://github.com/AMYPAD/NiftyPAD), and allows for multi-platform usage. The modular setup makes adding new functionalities easy, and the package is lightweight with minimal dependencies, making it easy to use and integrate into existing processing pipelines.

**Supplementary Information:**

The online version contains supplementary material available at 10.1007/s12021-022-09616-0.

## Introduction

As the biomedical research community is moving towards earlier disease detection, it has become increasingly relevant to capture subtle changes in biological processes Guo et al. (2020), Cohen and Klunk (2014). A technique that allows for both visualising and quantifying these processes *in vivo* is Positron Emission Tomography (PET) Guo et al. (2020), Cohen and Klunk (2014). PET scans can be used to obtain highly accurate, quantitative measurements, provided that a suitable scanning protocol is used. To this end, dynamic or more recently established dual-time window scanning protocols may be required, because of their ability to take into account physiological changes Bullich et al. (2018), Lammertsma (2017), Heeman et al. (2019), van Berckel et al. (2013). These protocols require longer scanning time, complex processing and pharmacokinetic analyses.

As the size of PET datasets continue to increase, so will the demand for efficient processing pipelines that provide high throughput Rabinovici et al. (2021), Ferrucci (2018), LaMontagne et al. (2019).

In addition to existing commercial software packages, such as PMOD (PMOD Technologies, Zurich, Switzerland), publicly available software packages for pharmacokinetic analysis of dynamic brain PET data have been introduced, including COMKAT Muzic and Cornelius (2001), PPET Boellaard et al. (2006), MIAKAT Gunn et al. (2016) (previously available), QModeling López-González et al. (2019), Magia Karjalainen et al. (2020b) and kinfitr Matheson (2019). PPET is written in IDL (L3Harris Geospatial Solutions, Inc.). COMKAT, MIAKAT, QModeling and Magia are written and run in MATLAB (The MathWorks, Inc.). Both IDL and MATLAB are not freely available. kinfitr is written in the freely available R language. In this work we present NiftyPAD, where PAD stands for *p*ackage for quantitative *a*nalysis of *d*ynamic PET data. It is written in Python, which is completely open source, and designed to increase the capacity of a pharmacokinetic modelling software package. An important feature NiftyPAD provides is the ability to analyse PET data acquired in a dual-time window protocol Heeman et al. (2019), to support the field given the growing number of studies that acquire early PET data after tracer injection in addition to the late PET scan LopesAlves et al. (2020), Cecchin et al. (2017), Son et al. (2020). A dual-time window protocol is useful for quantitative studies using tracers with slow kinetics because of their long dynamic acquisition times (up to 130 min). To decrease the scanning burden for the participant, a break is inserted in the middle of the scan during which the participant can leave the scanner and rest. The break also allows for interleaved acquisition, in which the scan of a second participant can be initiated during the break of the first participant. To allow for kinetic modelling, these dual-time window or dual-phase data require appropriate interpolation of the reference tissue curve, which has been rarely implemented in existing software packages Funck et al. (2018), Karjalainen et al. (2020a). Another novel feature of NiftyPAD is pharmacokinetic modelling through incorporation of ASL-derived relative perfusion measures for simultaneous PET-MR scans Scott et al. (2019). This feature circumvents the need for an early PET scan by combining a simultaneously acquired ASL scan with a static PET scan to allow for kinetic modelling.

The growing number of dynamic datasets and their complex analyses, demonstrate the need for software tools that facilitate straightforward and automated analyses of PET data. In addition, combining these aspects in an open-source software tool will improve the reproducibility of the results by allowing others to use the same algorithm, whilst maintaining the possibility of tailored software solutions. Therefore, NiftyPAD was designed to support several important features which are not available in other existing software packages for kinetic modeling:

- Freely available (Python-based) and open-source, allowing for full transparency
- Analysis of static, dynamic and dual-time window PET data
- Pharmacokinetic modelling with the incorporation of arterial spin labelling (ASL)-derived relative perfusion measures for simultaneous PET-MR scans
- Motion correction, through a built-in kinetics based realignment function, and options for excluding PET frames with large motion-misaligned attenuation correction in kinetic analysis

The present paper discusses features that are incorporated in the NiftyPAD software package, with focus on the modelling aspects. Implementation of NiftyPAD was evaluated by comparing its numerical results of estimating kinetic parameters with those obtained by using the established software packages PPET Boellaard et al. (2006) and QModeling López-González et al. (2019). Clinical PET data from various amyloid tracers were used for the evaluation. A selection of core models were assessed given the model availability in the existing software packages.

## Method

### NiftyPAD Overview

**Fig. 1:**
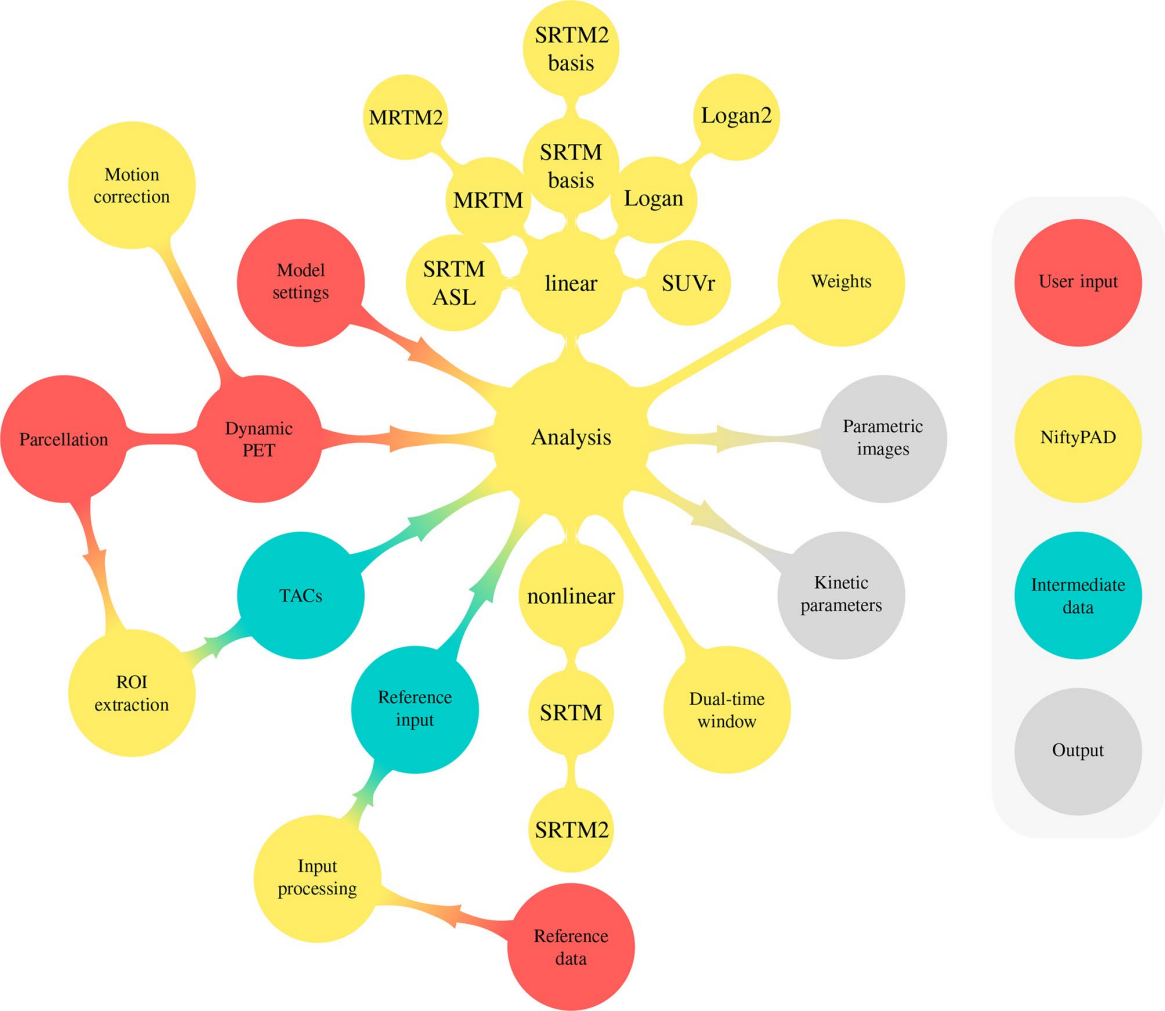
Features of NiftyPAD and its workflow. The features and functions NiftyPAD provides are shaded in  , including optional motion correction, reference input processing, a group of kinetic modelling methods and weighting schemes for analysing dynamic PET data. The required user inputs are shaded in  , the intermediate data in  , and the resulting outcome measures in

Figure 1 summarises the features of NiftyPAD and its workflow. NiftyPAD provides a group of reference-based kinetic models to generate parametric images or regional kinetic parameters. For quantification, NiftyPAD requires the user to provide a PET scan, data for obtaining a reference tissue time-activity curve (TAC), a region of interest (ROI) template in case of regional analyses, and modelling settings for the selected kinetic models. More specifically, reference tissue input processing is implemented for interpolating missing reference tissue data points of PET data acquired according to a dual-time window protocol and to improve accuracy of the reference tissue curve in case of noise or motion. Other implemented features include support for weighting the temporal data points and options for motion correction.

### User Input

As shown in Fig. 1, NiftyPAD requires a user to provide: 1) PET imaging data (static, dynamic or dual-time window) for parametric analysis, in combination with frame start and end times in seconds, 2) a ROI template (parcellation) for extracting regional TACs, or user-pre-defined TACs for regional analyses, 3) reference tissue input data, provided directly by the user or using NiftyPAD to extract for a named reference region, and 4) model settings to select the quantification methods and configurations of model parameters.

With regard to the PET scan, most commonly used imaging data formats are supported, such as dicom, nifti, analyze and ecat (see the NiBabel Python library for a full list of supported file types, https://doi.org/10.5281/zenodo.4295521). In addition, fitting settings (e.g. the number of basis functions and the applied boundary conditions) can be specified.

### Input Processing

NiftyPAD allows for processing of the reference tissue input data. This can be used to improve accuracy in case of noise or motion, or to interpolate missing data in the specific case of dual-time window scans where the dynamic data are acquired in separate sessions.

The code for this feature is available at https://github.com/AMYPAD/NiftyPAD/blob/master/niftypad/tac.py.

The following four methods for reference input processing are provided in NiftyPAD:

#### Linear Interpolation

The reference input is interpolated using linear functions, this is usually suitable for standard scan protocols without dual-time window.

#### Cubic Interpolation

The reference input is interpolated using cubic functions, when linear functions are not suitable for interpolating between two time points in the reference data.

#### Exponential Interpolation

The decay part of the reference input is interpolated using an exponential function:

1 $$\begin{aligned} C_R(t) = a_0 + a_1 e^{-b_1 t}, \;\;\;\; t \in [t_s, t_e], \end{aligned}$$

where $C_R(t)$ is the tracer concentration in the reference region, $a_0$, $a_1$, $b_1$ are the unknown parameters to be determined by the reference input data, $t_s$ and $t_e$ are the starting and ending times for the exponential decay phase chosen by the user.

#### Feng’s Plasma Input Model + 1TC

In addition to cubic and exponential interpolations, NiftyPAD also provides a compartmental model-based method for reference input processing. The tracer behaviour in the reference tissue is described by a one-tissue-compartment (1TC) model, with the operational function

2 $$\begin{aligned} C_R(t) = a_3 C_p(t) \otimes e^{-b_3 t}, \end{aligned}$$

where $C_R(t)$ and $C_p(t)$ are tracer concentrations in the reference tissue and arterial plasma, respectively, $a_3$ and $b_3$ are the unknown parameters to be determined by the reference input data. The plasma input function $C_p(t)$ is described by Feng’s plasma input model Feng et al. (1994) as

3 $$\begin{aligned} C_p(t) = (a_0 t - a_1- a_2 ) e^{-b_0 t} + a_1 e^{-b_1 t} + a_2 e^{-b_2 t}, \end{aligned}$$

where $a_0$, $a_1$, $a_2$, $b_0$, $b_1$, $b_2$ are unknown parameters determined together with $a_3$ and $b_3$ by fitting Equation 2 to the reference input data.

Examples of using cubic and exponential interpolations, and the Feng’s input model + 1TC method on clinical data are shown in “Comparison with Established Softwares”.

### Pharmacokinetic Quantification

The current version of NiftyPAD focuses on a group of reference tissue input based methods that do not require invasive arterial blood sampling or processing, which are widely applied to a range of neuro studies LaMontagne et al. (2019), Golla et al. (2016), Mertens et al. (2020).

The code for all methods is available at https://github.com/AMYPAD/NiftyPAD/blob/master/niftypad/models.py. The kinetic parameters of interest are estimated by numerical optimisation, using weighted (unweighted if weights are not given) least squares as the objective function.

For quantification of both region of interest (ROI) and voxel data, the following linear and nonlinear models are implemented in NiftyPAD:

#### Nonlinear Models

##### SRTM and SRTM2

The simplified reference tissue model SRTM Lammertsma and Hume (1996), and SRTM2 Wu and Carson (2002) with pre-defined tissue-to-plasma clearance $k'_2$ of the reference tissue are implemented with the operational functions

4 $$\begin{aligned} C_T(t) = R_1C_R(t) + [k_2 - R_1k_{2a}] C_R(t) \otimes e^{-k_{2a} t}, \end{aligned}$$

and

5 $$\begin{aligned} C_T(t) = R_1C_R(t) + R_1 [k'_2 - k_{2a}] C_R(t) \otimes e^{-k_{2a} t} \end{aligned}$$

respectively. Where $C_T(t)$ and $C_R(t)$ are tracer concentrations in the target and reference tissue compartment, respectively, $R_1$ is the relative tracer delivery parameter ($K_1$/$K_1'$), $k_2$ is the rate constant for transfer from free to plasma compartment, $k_{2a}$ is the apparent rate constant for transfer from the specific compartment to plasma and *t* is the time in minutes. The kinetic parameters of interest $R_1$, $k_2$, and $k_{2a}=k_2/(1+BP_{ND})$ are estimated by nonlinear optimisation. The non-displaceable binding potential, $BP_{ND}$, is then derived by $BP_{ND}=k_2 /k_{2a}-1$ for SRTM, and $BP_{ND}=R_1k'_2 /k_{2a}-1$ for SRTM2.

#### Linear Models

##### SRTM Basis and SRTM2 Basis

The linearisation of SRTM with basis functions is implemented based on Gunn et al. (1997), with the operational function

6 $$\begin{aligned} C_T(t) = R_1C_R(t) + \theta B^i(t), \end{aligned}$$

where $\theta B^i$ are the number of basis functions and the basis functions are calculated as

7 $$\begin{aligned} B^{i}(t) = C_R(t) \otimes e^{-k^{i}_{2a} t}. \end{aligned}$$

The pre-defined $k'_2$ version SRTM2 with basis functions is implemented based on Wu and Carson (2002), with the operational function

8 $$\begin{aligned} C_T(t) = R_1 B^{i}_{k'_2}(t), \end{aligned}$$

where the basis functions are calculated as

9 $$\begin{aligned} B^{i}_{k'_2}(t) = C_R(t) + (k'_2 - k^{i}_{2a}) C_R(t) \otimes e^{-k^{i}_{2a}t}. \end{aligned}$$

Details on solving the linearised problem and calculations of the kinetic parameters of interest $R_1$, $k_2$ and $BP_{ND}$ can be found in Gunn et al. (1997) and Wu and Carson (2002).

##### SRTM ASL

NiftyPAD features a recently developed model SRTM ASL Scott et al. (2019) for analysing PET data acquired by a simultaneous PET-MR scanner where arterial spin labelling (ASL) is available to provide the perfusion information and derive the relative influx rate $R_1$. In SRTM ASL, $R_1$ is derived from ASL MRI data by using Equations 3 and 4 in Scott et al. (2019). With pre-determined $R_1$, the SRTM basis model can be re-written as

10 $$\begin{aligned} C^{d}_T(t) = C_T(t) - R_1C_R(t) = \theta B_i(t), \end{aligned}$$

where $C^{d}_T(t)$ is a dummy variable determined before fitting. $\theta$ is then solved with the pre-defined basis functions $B_i(t)$, which follows the same definition as in Eq. 7. The kinetic parameters of interest $k_2$ and $BP_{ND}$ are then derived as in Scott et al. (2019).

##### Logan and Logan2

The graphical analysis methods with reference input Logan plot, and Logan2 with pre-defined $k'_2$ are both implemented based on Logan et al. (1996), by

11 $$\begin{aligned} \frac{\int ^{T}_0 C_T(t) dt}{C_T(T)} = DVR \frac{\int ^{T}_0 C_R(t) dt}{C_T(T)} + int', \end{aligned}$$

and

12 $$\begin{aligned} \frac{\int ^{T}_0 C_T(t) dt}{C_T(T)} = DVR \frac{\int ^{T}_0 C_R(t) dt + C_R(t)/k'_2}{C_T(T)} + int' \end{aligned}$$

respectively, where $int'$ stands for the intercept in the linear regression, *DVR* for the distribution volume ratio, and binding potential $BP_{ND}=DVR-1$.

##### MRTM and MRTM2

The Ichise’s Multilinear Reference Tissue Model MRTM and MRTM2 are both implemented based on Ichise et al. (2003). The operational equations for MRTM and MRTM2 with pre-defined $k'_2$ are

13 $$\begin{aligned} C_T(T) = \gamma _1\int ^{T}_0 C_R(t) dt + \gamma _2\int ^{T}_0 C_T(t) dt + \gamma _3 C_R(T), \end{aligned}$$

and

14 $$\begin{aligned} C_T(T) = \gamma _1(\int ^{T}_0 C_R(t) dt + \frac{1}{k'_2} C_R(T) )+ \gamma _2\int ^{T}_0 C_T(t) dt \end{aligned}$$

respectively, where the coefficients $\gamma _1$, $\gamma _2$ and $\gamma _3$ are determined by linear regression. The kinetic parameter of interest $BP_{ND}$ is then calculated by $BP_{ND}=-(\gamma _1/\gamma _2+1)$.

**SUVr**

Standardised uptake value ratio (SUVr), the most commonly used semi-quantitative method is calculated as the ratio of target to reference tissue activity for a pre-defined time window.

### Weighting Schemes

To improve the accuracy of kinetic analysis, weighting schemes are commonly used when fitting a kinetic model to dynamic PET data. Introduction of weighting schemes can improve model fitting especially with noisy PET data, or data acquired using a dual-time window protocol. NiftyPAD therefore provides a number of weighting schemes to facilitate optimal fitting Yaqub et al. (2006), and also allows the user to input pre-determined weights.

The code for this feature is available at https://github.com/AMYPAD/NiftyPAD/blob/master/niftypad/weight.py.

### Motion Correction

NiftyPAD features an integrated motion correction module, which can be used with PET imaging data. The module consists of two features: 1) exclude PET frames that suffer from severe motion and cause a mismatch in attenuation correction, and 2) correct for motion between PET frames by performing a groupwise registration that minimises the errors in the fits of the pharmacokinetic modelling. This approach is implemented based on Jiao et al. (2014). Note that for dual-time windows scans, this motion correction approach can be applied before aligning the early and late frames. However the co-registration of the early and late frames using external structural images can greatly improve the motion estimation and correction.

The code for the this feature is available at https://github.com/AMYPAD/NiftyPAD/blob/master/niftypad/image_process/motion_correction.py.

## Evaluation

Performance of the main functions provided in NiftyPAD was evaluated using clinical data. Here, we present the results of 1) processing the reference tissue input data for dual-time window scans with various methods, 2) comparing the kinetic parameters computed by NiftyPAD and established software packages, 3) applying the ASL SRTM method to a dynamic dataset, and 4) generating parametric images.

### Performance on Input Processing

**Fig. 2:**
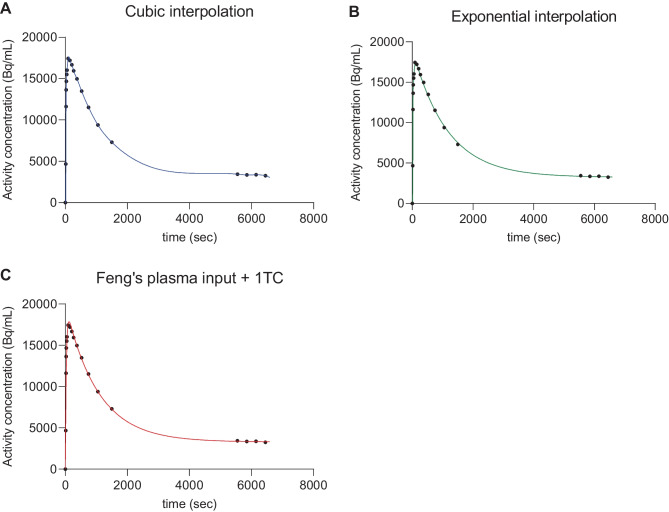
Interpolation of reference tissue TAC from one [^18^F]florbetaben scan with dual-time window acquisition, using **A** cubic interpolation, **B** exponential interpolation, and **C** Feng’s plasma input + 1TC model. Reference tissue input processing serves as an essential first step before kinetic analysis of dual-time window PET scans

For PET scans acquired using dual-time window protocols, appropriate reference tissue input interpolation of missing data-points between the two parts of a scan is an essential step before performing kinetic analysis, as linear interpolation or simply concatenating the two parts of the scan does not follow the kinetics in the reference data. Figure 2 shows an example of the interpolated reference tissue TACs obtained by using cubic interpolation, exponential interpolation and Feng’s plasma input + 1TC model, which are available in the NiftyPAD software package and described in “Input Processing”.

### Comparison with Established Softwares

As the core function of NiftyPAD is performing kinetic analysis on dynamic PET data, we evaluated the implementation of the kinetic models SRTM basis, SRTM2 basis, Logan and MRTM2, by comparing the kinetic parameters computed by NiftyPAD with those computed by the two established softwares, PPET Boellaard et al. (2006) and QModeling López-González et al. (2019). Given the model availability and implementation similarity, PPET was used for the graphical models Logan and MRTM2, and QModeling was used for the basis function based methods SRTM basis and SRTM2 basis. We did not use PPET for the SRTM models as the computation of the basis functions and the solution of the linear problem were implemented differently in PPET.

Clinical data from eight subjects scanned with four different amyloid tracers were used for this evaluation. The subjects include A$\beta$-positives and A$\beta$-negatives. Four were from the AMYPAD Prognostic and Natural History Study (PNHS) in which two were scanned with [^18^F]flutemetamol and two with [^18^F]florbetaben LopesAlves et al. (2020). Two other subjects were scanned with [^18^F]florbetapir Golla et al. (2019), and the remaining two were part of a test-retest or longitudinal [^11^C]PiB dataset Tolboom et al. (2009), Ossenkoppele et al. (2012). All participants provided written informed consent before participating in the study. The local Medical Ethics Review Committee approved the study protocol and scans were acquired at the Amsterdam UMC, location VUmc. The full demographic information of the subjects can be found in Table 1 in Supplementary Materials.

Detailed acquisition and processing of these scans are described in LopesAlves et al. (2020), Heeman et al. (2020), Verfaillie et al. (2021). Notably, the [^18^F]flutemetamol and [^18^F]florbetaben scans followed the dual-time window protocol. These scans were not included in the comparison of NiftyPAD and QModeling, as the latter one does not support data acquired according to a dual-time window protocol.

**Fig. 3:**
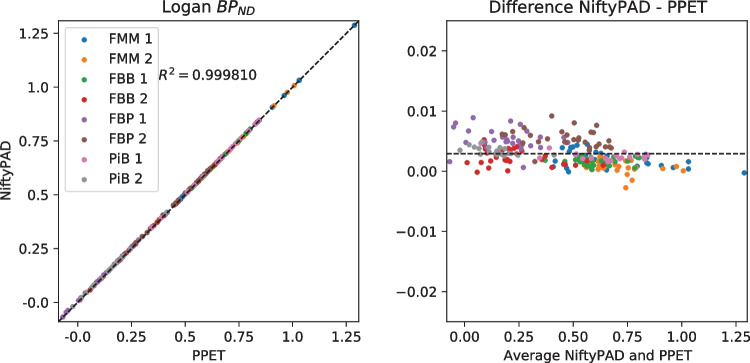
Correlation and Bland-Altman plots of the $BP_{ND}$ values computed by NiftyPAD and PPET using the Logan reference model. Data points correspond to different brain regions from each subject. The dashed lines are the line of identity on the left, and the mean difference on the right

**Fig. 4:**
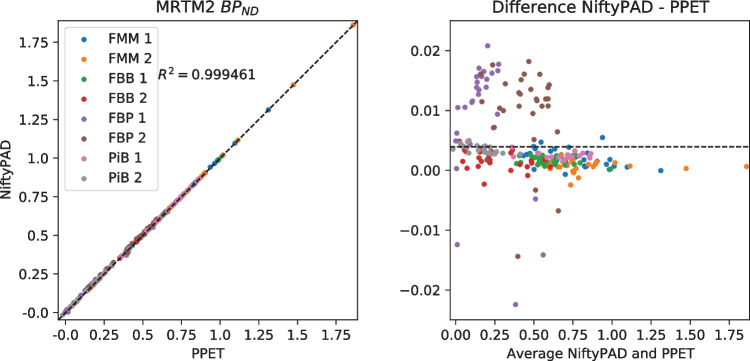
Correlation and Bland-Altman plots of the $BP_{ND}$ values computed by NiftyPAD and PPET using the MRTM2 model. Data points correspond to different brain regions from each subject. The dashed lines are the line of identity on the left, and the mean difference on the right

**Fig. 5:**
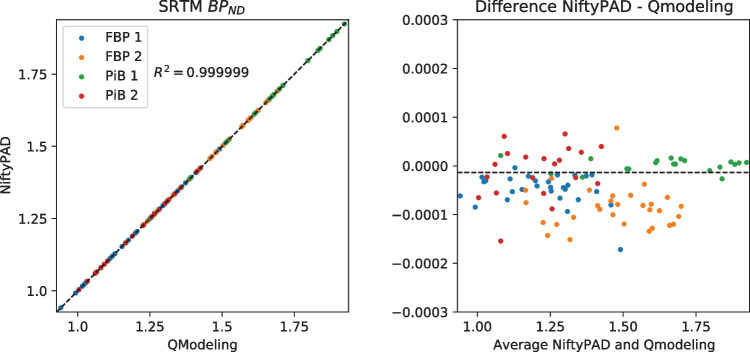
Correlation and Bland-Altman plots of the $BP_{ND}$ values computed by NiftyPAD and QModeling using the SRTM model with basis functions. Data points correspond to different brain regions from each subject. The dashed lines are the line of identity on the left, and the mean difference on the right

**Fig. 6:**
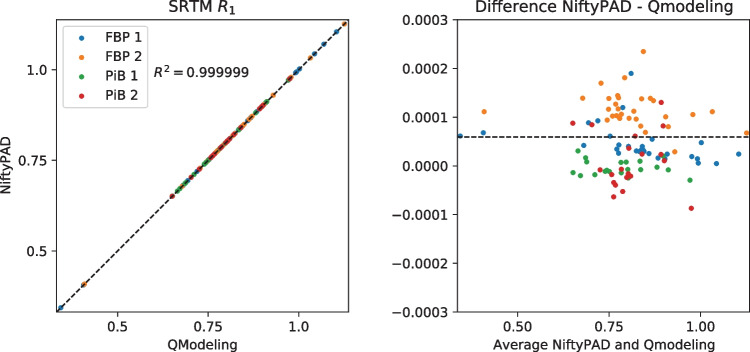
Correlation and Bland-Altman plots of the $R_1$ values computed by NiftyPAD and QModeling using the SRTM model with basis functions. Data points correspond to different brain regions from each subject. The dashed lines are the line of identity on the left, and the mean difference on the right

**Fig. 7:**
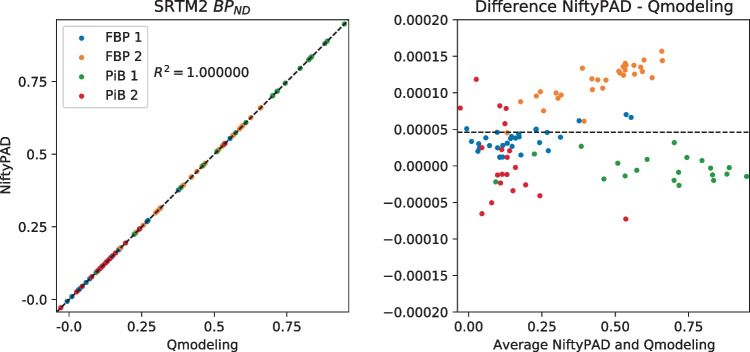
Correlation and Bland-Altman plots of the $BP_{ND}$ values computed by NiftyPAD and QModeling using the SRTM2 model with basis functions. Data points correspond to different brain regions from each subject. The dashed lines are the line of identity on the left, and the mean difference on the right

**Fig. 8:**
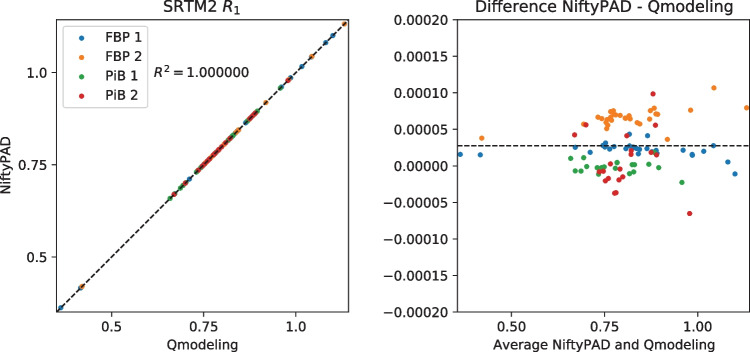
Correlation and Bland-Altman plots of the $R_1$ values computed by NiftyPAD and QModeling using the SRTM2 model with basis functions. Data points correspond to different brain regions from each subject. The dashed lines are the line of identity on the left, and the mean difference on the right

For each scan, brain parcellation was applied to extract the time-activity curves (TACs) from anatomical regions of interest. Thirty regional TACs were selected from each subject with a range of volume sizes and binding levels. These regional TACs were used to quantitatively compare the kinetic parameter values estimated by NiftyPAD and the established softwares. Correlation and Bland-Altman plots of the kinetic parameters obtained by using NiftyPAD and the reference software are shown in Figs. 3, 4, 5, 6, 7 and 8.

Comparing with PPET for the graphical models, the absolute differences were in the order of $0-10^{-2}$ for $BP_{ND}$. This can be due to the difference in precision between the programming languages, as PPET is written in IDL and has by default single precision, whereas NiftyPAD is written in Python and has by default double precision. Also the implementation of the linear regression used in the graphical models can results in differences between these two packages. Comparing with QModeling for the basis function based SRTM models, the absolute differences were in the order of 0-$10^{-4}$ for $BP_{ND}$ and $R_1$. We did not find differences in implementation between NiftyPAD and QModeling of these two SRTM models. The differences are considered to be caused by the difference in handling the floating point calculations between Python and MATLAB which QModeling uses.

### Application of SRTM ASL to Regional Data

In order to show the effectiveness of the SRTM ASL model for estimating $BP_{ND}$ from a shorter acquisition, the model was applied to TACs from one dynamic [^18^F]flutemetamol data set that had been acquired using a dual-time window protocol with an acquisition phase from 0-30 minutes and 90-110 minutes post injection (A$\beta$-negative, male, 63.0 years old). The scan was acquired using a Siemens mMR Biograph and, simultaneously, a T1 MPRAGE Sagittal MRI was acquired. The T1-weighted MR image was segmented into 11 different regions (white matter, cortex, cerebellar white matter, thalamus, caudate, putamen, pallidum, brain stem, hippocampus, amygdala and accumbens) using Freesurfer version 6.0.0 Fischl (2012), and TAC data were extracted from the PET scan for each region. Note that for the purpose of code validation, we did not use the $R_1$ derived from ASL MRI as in Scott et al. (2019), for the reason that the noise in ASL will propagate into the derived $R_1$ and the evaluation of the SRTM ASL implementation will be confounded. Instead, SRTM basis was applied to the full PET data to determine $R_1$ and then, SRTM ASL was applied to the 90-110 minutes data with the determined $R_1$ as an input parameter varied per region. The $BP_{ND}$ values from SRTM ASL and SRTM basis were compared using linear regression analyses.

**Fig. 9:**
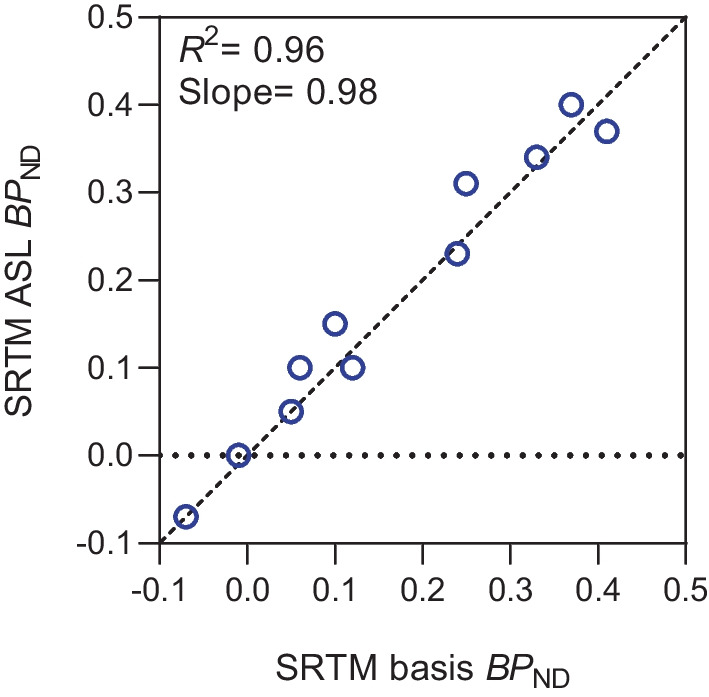
Relationship between SRTM basis and SRTM ASL derived $BP_{ND}$. Correlation between $BP_{ND}$ derived from two different methods, with $R^2$ and slope parameters corresponding to a linear regression analysis. Dashed line corresponds to the line of identity

Excellent correlation and negligible bias was observed between the SRTM basis and SRTM ASL methods (Fig. 9). The $BP_{ND}$ values from SRTM ASL and SRTM basis are not identical due to the difference in the length of scan involved in analysis, in this case 0-110 minutes for SRTM basis and 90-110 minutes for SRTM ASL.

### Parametric Image Generation

**Fig. 10:**
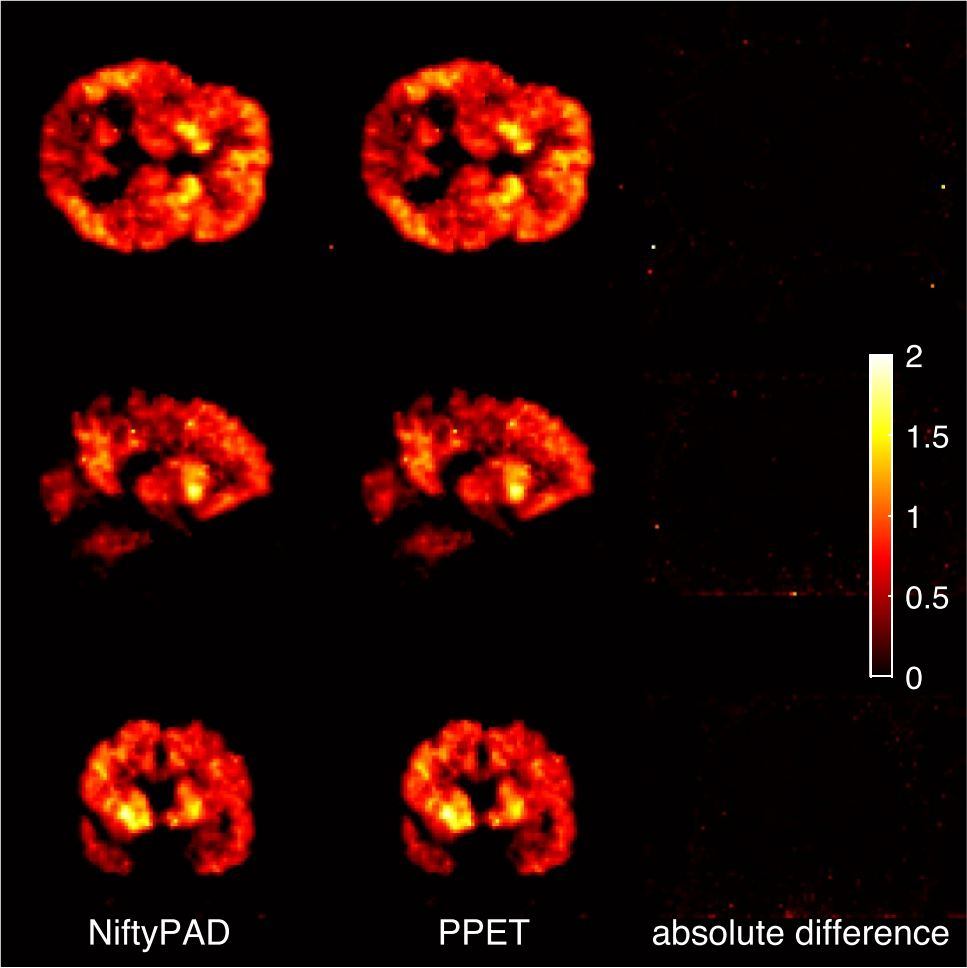
Parametric $BP_{ND}$ images from a [^11^C]PiB scan derived by the Logan reference model using NiftyPAD and PPET

One scan of dynamic [^11^C]PiB data of 90 minutes duration was used to illustrate the parametric image generation. The scan belongs to a patient with Alzheimer’s disease dementia (A$\beta$-positive, male, 60 years old) Tolboom et al. (2009). Pre-processing was done as described previously Heeman et al. (2020) and Logan reference model was used to generate parametric images with cerebellar grey matter as reference tissue. $BP_{ND}$ images derived using NiftyPAD and PPET are shown in Fig. 10.

## Discussion

In this paper we present NiftyPAD, a software package for performing kinetic analysis of dynamic PET images from clinical studies. It is written in Python, is open source and freely available, thus providing full transparency. The software is multi-platform, stand-alone, has minimal dependencies, all of which are standard Python packages, and is therefore easy to be extended and integrated into any PET data processing and analysis pipeline. The key novelties of NiftyPAD includes the analyses of dual-time window scans with reference input processing, and pharmacokinetic modelling with shortened PET acquisitions through the incorporation of arterial spin labelling (ASL)-derived relative perfusion measures.

Results produced by NiftyPAD were compared with those produced by two established software packages PPET and QModeling. NiftyPAD achieved reliable results and we noticed that differences in the programming languages used, and especially differences in the implementation of the numerical operations underlying the kinetic models can result in differences in the final outcome measures. For data consistency, we recommend to use the same kinetic analysis software to reduce the variability when comparing kinetic parameters.

Compared with currently available pharmacokinetic modelling software packages, a major novelty of NiftyPAD is the ability to analyse PET data acquired in a dual-time window protocol. This feature is of great importance to the field, given the growing number of studies that acquire early PET data at tracer injection in addition to a late, static scan. More specifically, to allow for kinetic modelling, these dual-time window data require interpolation of the missing data points of the reference tissue curve, which can be done via reference input processing. Of course, as each tracer has distinct kinetics, the optimal interpolation scheme requires validation per tracer. Another distinct feature of NiftyPAD is pharmacokinetic modelling through incorporation of ASL-derived relative perfusion measures for simultaneous PET-MR scans. This feature circumvents the need for an early PET scan by combining relative perfusion measures from an ASL scan with a late PET scan to allow for kinetic modelling. However, as this model has only been applied to [^18^F]florbetapir data in a proof-of-concept study, its applicability to other (amyloid) tracers is still warranted Scott et al. (2019). Finally, given that patient motion occurs both in clinical and research practise, NiftyPAD offers two distinct features to limit the effect of motion on quantification.

The current version of the NiftyPAD software package can be extended in several directions. For example, plasma input-based models, which are of particular interest for tracer-validation studies can be added to broaden the analysis capacity. At present, documentation, demos/tutorials and graphical user interface are under development in order to facilitate the use of NiftyPAD in routine clinical studies. Lastly, future versions will focus more on quality assessment of the generated output, which is currently also under development.

## Conclusion

NiftyPAD is a freely available, open-source software program for quantitative PET analyses. It features unique modules that allow for analysing data acquired in a dual-time window protocol, optional kinetic based motion correction and incorporation of ASL derived relative perfusion measures into the pharmacokinetic modelling routine. Furthermore, the software has been shown to provide accurate estimates of pharmacokinetic parameters and can be successfully applied to PET data on a regional basis as well as voxel-by-voxel.

## Supplementary Information

Below is the link to the electronic supplementary material.

Supplementary file1 (PDF 115 kb)

## Data Availability

The presented software is open-source and freely available at https://github.com/AMYPAD/NiftyPAD.
