# Supplementary material for: NiftyPAD - Novel Python Package for Quantitative Analysis of Dynamic PET Data
Source: Neuroinformatics. 2023 Jan 9;21(2):457–68. doi: 10.1007/s12021-022-09616-0 (PMC10085912; doi:10.1007/s12021-022-09616-0)
Supplement: Supplementary file 1 — Supplementary file1 (PDF 115 kb) [file 12021_2022_9616_MOESM1_ESM.pdf]

Supplementary materials

Table 1 gives demographics of the eight subjects used in Sec. 3.2.

Table 1 Demographics of the eight subjects. FBB=[^{18}F]florbetaben, FMM=[^{18}F]flutemetamol, FBP=[^{18}F]florbetapir, CU=Cognitively Unimpaired. MMSE=Mini-Mental State Examination, A=Philips Ingenuity TF PET/MR, B=Siemens ECAT EXACT HR+ scanner, C=Philips Ingenuity TF PET/CT

Subject	Tracer	Diagnosis	Age	Sex	Visual read	MMSE	Scanner
1	FBB	CU	66	F	1	28	A
2	FBB	CU	68	F	0	30	A
3	FMM	CU	80	M	0	27	A
4	FMM	CU	69	F	1	30	A
5	PiB	AD dementia	60	M	1	21	B
6	PiB	CU	68	M	0	30	B
7	FBP	CU	63	M	0	29	C
8	FBP	AD dementia	71	M	1	25	C
